# Understanding host immune responses in *Clostridioides difficile* infection: Implications for pathogenesis and immunotherapy

**DOI:** 10.1002/imt2.200

**Published:** 2024-05-11

**Authors:** Lamei Wang, Javier A. Villafuerte Gálvez, Christina Lee, Shengru Wu, Ciaran P. Kelly, Xinhua Chen, Yangchun Cao

**Affiliations:** ^1^ College of Animal Science and Technology Northwest A&F University Yangling China; ^2^ Division of Gastroenterology, Department of Medicine, Beth Israel Deaconess Medical Center Harvard Medical School Boston Massachusetts USA

**Keywords:** antibody, *Clostridium difficile*, gut microbiota, host immune, toxins

## Abstract

*Clostridioides difficile* (*C. difficile*) is the predominant causative agent of nosocomial diarrhea worldwide. Infection with *C. difficile* occurs due to the secretion of large glycosylating toxin proteins, which can lead to toxic megacolon or mortality in susceptible hosts. A critical aspect of *C. difficile's* biology is its ability to persist asymptomatically within the human host. Individuals harboring asymptomatic colonization or experiencing a single episode of *C. difficile* infection (CDI) without recurrence exhibit heightened immune responses compared to symptomatic counterparts. The significance of these immune responses cannot be overstated, as they play critical roles in the development, progression, prognosis, and outcomes of CDI. Nonetheless, our current comprehension of the immune responses implicated in CDI remains limited. Therefore, further investigation is imperative to elucidate their underlying mechanisms. This review explores recent advancements in comprehending CDI pathogenesis and how the host immune system response influences disease progression and severity, aiming to enhance our capacity to develop immunotherapy‐based treatments for CDI.

## Highlights

- The progression of *Clostridioides difficile* infection (CDI) is influenced by various factors, including the use of antibiotics, age, comorbidities, immune gene polymorphisms, and antibody levels.
- Immune response plays a critical role in controlling both the course and severity of CDI.
- Immunotherapy holds significant promise for advancing both treatment and prevention strategies for CDI. This encompasses the application of monoclonal antibodies, vaccines, and fecal microbiota transplantation (FMT).

## Graphical abstract

The progression of *Clostridioides difficile* infection (CDI) is shaped by multiple factors, such as antibiotic usage, age, comorbidities, immune gene polymorphisms, and antibody levels. Among these, the immune response stands out as a pivotal determinant in controlling both the course and severity of CDI. Leveraging immunotherapy presents substantial promise for advancing treatment and prevention strategies for CDI. This encompasses a range of approaches, including the use of monoclonal antibodies targeting *C. difficile* toxins, the development of vaccines to stimulate protective immune responses, and the application of fecal microbiota transplantation to restore microbial balance and bolster colonization resistance against *C. difficile*. These innovative immunotherapeutic interventions hold the potential to revolutionize CDI management by targeting key aspects of the disease's pathogenesis and immune regulation.

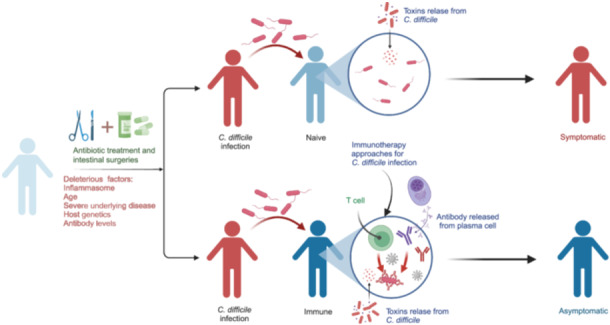

## INTRODUCTION

*Clostridioides difficile* (*C. difficile*) is an anaerobic, gram‐positive bacillus capable of sporulation. It thrives in the human gut, especially following disruptions to the natural colonic microbiota caused by antibiotic usage. This pathogen can cause severe gastrointestinal infections in humans, ranging from mild diarrhea to fulminant colitis and even death [1]. In the United States, it results in significant healthcare expenditures [2, 3]. *C. difficile* infection (CDI) presents a considerable global public health challenge, with approximately 500,000 cases and around 20,000 deaths reported annually worldwide [4]. Over recent decades, there has been a notable increase in both incidence and severity of CDI, particularly within healthcare settings. Vulnerable populations, such as elderly individuals, hospitalized patients with a history of extensive antibiotic use or weakened immune systems, are at elevated risk of developing CDI.

CDI occurs when the *C. difficile* colonizes the gut and releases toxins that damage the intestinal mucous membrane and actin cytoskeleton. The pathogenesis of CDI involves several key steps. Initially, colonization occurs following the ingestion of *C. difficile* spores, typically coinciding with antibiotic therapy, wherein the spores can withstand the acidic milieu of the stomach and transit to the colon. Under specific conditions, such as reduced competition from the natural gut microbiota, these spores undergo germination into vegetative cells upon entering the colon [5]. Subsequently, *C. difficile* releases two primary toxins known as enterotoxin A (TcdA) and cytotoxin B (TcdB), which bind to receptors on intestinal cells, disrupting cellular function and triggering an inflammatory response and diarrhea [1]. The significance of impairing cellular function by TcdA and TcdB underscores their role in the pathology of CDI and emphasizes the importance of targeting these toxins in therapeutic interventions.

The gastrointestinal microbiome plays a crucial role in protecting against CDI by competing with *C. difficile* for resources and producing metabolites that inhibit its growth. When broad‐spectrum antibiotics disturb this balance, it creates an environment favorable to *C. difficile* proliferation. Additionally, antibiotics can eliminate beneficial bacteria that help maintain gut health, further exacerbating CDI and fostering its recurrence. Recurrent CDI poses a significant clinical challenge due to factors such as persistent spores within the gut and the disruption of the gastrointestinal microbiome caused by broad‐spectrum antibiotics [6]. Hence, there is a pressing need to investigate novel alternative therapies for managing CDI. While existing treatment modalities demonstrate efficacy, challenges persist, especially regarding the management of recurrent CDI. Ongoing research is exploring alternative therapeutic avenues, including probiotics, phage therapy, antitoxin therapy, antimicrobial peptides, and immunomodulatory strategies. Furthermore, emerging technologies like gene editing and other biotechnological approaches hold promise in revolutionizing CDI treatment. In this context, our focus lies on exploring immunomodulatory strategies for CDI treatment, aiming to identify more effective and safer therapeutic options to tackle this clinical challenge.

**Figure 1:**
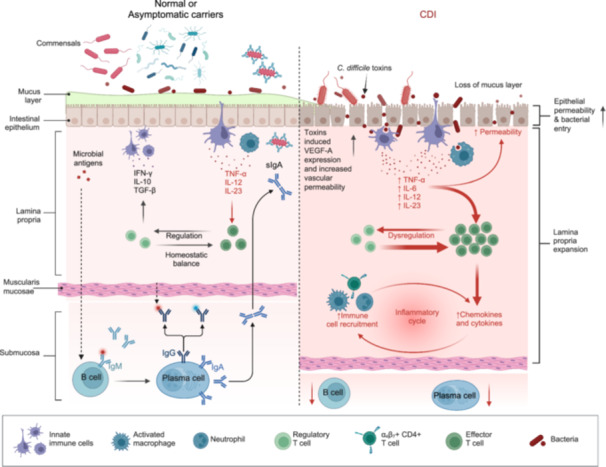
The intestinal immune response in health and CDI varies significantly between healthy individuals and those affected by CDI. In healthy individuals (left panel), the lamina propria typically hosts a diverse array of immune cells and secreted cytokines. Anti‐inflammatory mediators, such as TGF‐β and IL‐10, play crucial roles in suppressing immune responses. Additionally, both innate and adaptive immune cells release pro‐inflammatory mediators that regulate the entry of intestinal microbiota and provide defense against pathogens. Levels of IgA, IgM, and IgG are generally higher in healthy individuals compared to those afflicted with CDI. In individuals experiencing intestinal inflammation (right panel), various factors contribute to heightened bacterial exposure. These factors include disruption of the protective mucus layer, dysregulation of tight junctions in the epithelial cells, increased permeability of the intestinal lining, and enhanced adherence of bacteria to the epithelial cells. In cases of CDI, innate immune cells demonstrate heightened production levels of TNF‐α, IL‐6, IL‐12, IL‐23, and chemokines. *C. difficile*, *Clostridioides difficile*; CDI, *C. difficile* infection; TGF‐β, transforming growth factor‐beta; IL‐10, interleukin‐10; IgA, immunoglobulin A; IgM, immunoglobulin M; TNF‐α, tumor necrosis factor‐alpha; sIgA, secretory immunoglobulin A; VEGF‐A, vascular endothelial growth factor A; IFN‐γ, interferon‐gamma.

The immune response plays a critical role in combating CDI, as the intestinal mucosal immune system serves as the primary defense mechanism against this infection. During CDI, toxins produced by *C. difficile* induce an inflammatory reaction in the intestinal mucosa, thereby activating host immune cells and releasing immune factors (Figure 1). Macrophages, lymphocytes, and dendritic cells (DCs) are among the important immune cells involved in detecting and eliminating harmful bacteria and toxins to protect the host [7]. Notably, the absence of innate lymphoid cells (ILCs) can significantly increase mortality during the acute phase. Studies have shown that transferring CD90^+^ and CD127^+^ ILCs to ILC‐deficient mice, which exhibit high susceptibility to CDI, promotes their recovery. Despite the persistent proliferation of *C. difficile* and TcdA and B production, the mice were able to gain weight and resolve diarrhea [8]. Furthermore, the regulation of the immune system and inflammation can impact clinical manifestations, such as disease severity and the likelihood of recurrence in CDI patients. Studies have revealed that individuals with recurrent CDI tend to exhibit lower levels of IgM against TcdA and B compared to those experiencing initial infections [9]. Despite this observation, the precise mechanisms governing the interplay between *C. difficile* and immune function remain unclear. Attaining a comprehensive understanding of the immune response to *C. difficile* in both noninflammatory (homeostatic) and inflammatory conditions will greatly enhance our ability to effectively manage and prevent disease progression.

## PATHOGENESIS OF CDI

The pathology of CDI primarily arises from the activity of two homologous toxins, TcdA and TcdB. These toxins bind to receptors on host cells and subsequently undergo endocytosis by the host cells. Both TcdA and TcdB possess domains capable of directly impairing the Rho/Rac GTPases of the host through glycosylation, resulting in functional impairment of the cell. This impairment disrupts the integrity of the colonic epithelium by causing breakdown of the actin cytoskeleton and compromising the barrier function of epithelial cells, ultimately leading to subsequent apoptosis and tissue damage. Damage to host epithelial cells triggers increased vascular permeability, which in turn facilitates the release of erythrocytes and heme into the gastrointestinal lumen, ultimately leading to diarrhea and even colitis. Furthermore, certain strains of *C. difficile* produce a binary toxin (CDT), which exacerbates CDI severity by harming host cells [10].

The dysbiosis of gut microbiota plays a pivotal role in the pathogenesis of CDI. The presence of *C. difficile* toxins disrupts the homeostasis of commensal bacteria in the gastrointestinal tract, leading to inflammation and perturbation of diverse metabolic pathways, including carbohydrate and amino acid absorption and utilization by host cells. This disruption creates an unfavorable milieu that facilitates colonization and proliferation of *C. difficile*. Dysbiosis not only promotes the survival of *C. difficile* but also significantly impacts immune function in the gut. Comparative analysis of the microbiomes of CDI patients and asymptomatically colonized individuals reveals significant differences. Asymptomatically colonized patients exhibit a significant enrichment of species belonging to the class *Clostridia* in their microbiomes. Furthermore, their microbiomes show an enrichment of sucrose degradation pathways carried by commensal *Clostridia*, alongside glycoside hydrolases likely involved in starch and sucrose degradation. Fecal metabolomics analysis further supports this carbohydrate degradation profile by identifying an abundance of carbohydrate compounds in asymptomatically colonized patients compared to CDI patients [11].

Moreover, dysbiosis induced by *C. difficile* toxins can impair immune function in the gut by compromising the protective role of beneficial bacteria and weakening the immune response against pathogens like *C. difficile*. This disruption affects the gut‐associated lymphoid tissue (GALT) and mucosal immune system, leading to reduced production of antimicrobial peptides, immunoglobulins (Ig)s, and other protective factors. Consequently, the mucosal immune defense weakens, creating an environment that facilitates the more effective establishment of CDI [12]. The delicate balance between the host immune system and the gut microbiota is crucial for maintaining gut homeostasis and preventing infection. Disruption of this balance by *C. difficile* toxins not only contributes to the initial infection but also promotes recurrent CDI.

**Figure 2:**
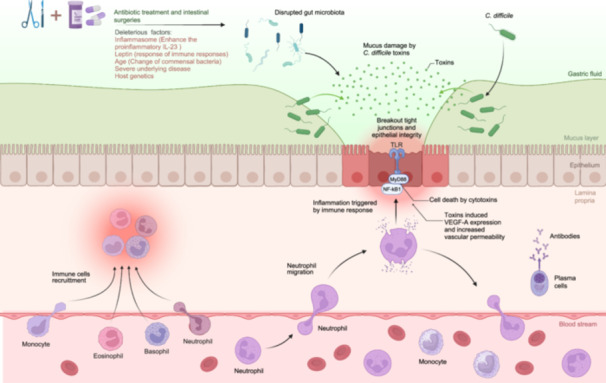
Pathogenesis of CDI. Prolonged antibiotic usage and intestinal surgeries can disturb the gut microbiota, thus potentially contributing to CDI. Risk factors linked to the advancement of CDI encompass age‐related alterations in commensal bacteria, underlying comorbidities, and host genetics. NF‐κB, nuclear factor‐kappa B; TLRs, Toll‐like receptors.

The progression and incidence of CDI are influenced by a multitude of factors, all of which can significantly impact the development and severity of the infection, such as antibiotic usage and intestinal surgeries, advanced age, comorbidities causing functional impairment, genetic variations in immune genes (such as interleukin (IL)−8), and reduced levels of antibodies against toxins (Figure 2) [13]. Immunosuppressed individuals exhibit a notably higher prevalence of CDI compared to the general population. For instance, within the hematology‐oncology community, rates range from 6% to 33%, while lung transplant recipients experience rates as high as 23%. Among patients with human immunodeficiency virus, there is a rate of 7.1‐8.3 cases per 1000 patient‐years. Recurrence of CDI is also common among immunocompromised individuals, with rates reaching up to 40% in both the hematology‐oncology population and solid organ transplant recipients. Before the coronavirus disease 2019 (COVID‐19) pandemic, the estimated global incidence of healthcare‐associated CDI ranged from 2.8 to 15.8 cases per 10,000 patient‐days [14]. Additionally, research has indicated that individuals diagnosed with inflammatory bowel disease (IBD) are more susceptible to contracting CDI in comparison to those without IBD [15, 16]. Moreover, individuals who have contracted severe acute respiratory syndrome coronavirus 2 (SARS‐CoV‐2) and those who have recovered from COVID‐19 may be considered high‐risk patients for developing CDI [17, 18, 19]. Several factors contribute to this increased risk, including frequent use of antimicrobial agents during treatment for COVID‐19‐related complications and potential disruptions to the normal gut microbiota [20]. Moreover, the immune system may be compromised during the course of SARS‐CoV‐2 infection or as a result of postinfection complications, making individuals more susceptible to opportunistic infections such as CDI. Therefore, it is crucial for healthcare providers to be vigilant for signs and symptoms of CDI in patients with a history of COVID‐19 and to implement appropriate preventive measures and treatment strategies to mitigate the risk of CDI in this population.

Understanding these factors is crucial for developing effective strategies to prevent and treat CDI, particularly in vulnerable populations such as the immunocompromised and those with underlying health conditions.

## IMPORTANCE OF HOST IMMUNE RESPONSE TO TREAT *C. DIFFICILE*

**Figure 3:**
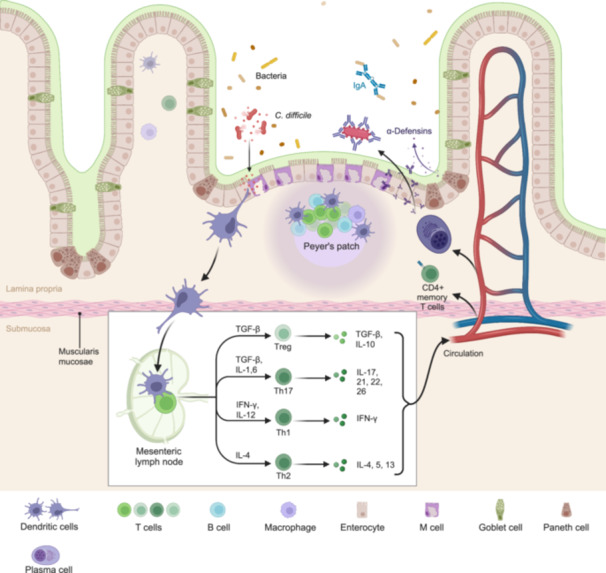
Intestinal immune system. In a healthy state, goblet cells secrete mucus to protect intestinal epithelial cells from bacterial exposure. Additionally, Paneth cells release antimicrobial peptides, including α‐defensins, while IgA is synthesized to enhance defense against luminal microbiota. DCs within secondary lymphoid organs, such as Peyer's patches and mesenteric lymph nodes, present antigens to naive CD4^+^ T cells. The differentiation of CD4^+^ T‐cell subgroups, including regulatory T cells (e.g., Treg) and Th cells (e.g., Th1, Th2, and Th17), is influenced by factors such as the phenotype of the antigen‐presenting cells and the cytokine milieu (e.g., TGF‐β and IL‐10). Moreover, enterotropic molecules are induced to facilitate the homing of lymphocytes from the systemic circulation to the gut. Once activated, CD4^+^ T cells migrate to the intestinal lamina propria to perform effector functions. DCs, dendritic cells; Th, T helper.

Immune cells play crucial roles in the development, progression, and prevention of CDI. They produce a variety of cytokines, including interferons (IFN)s, tumor necrosis factor (TNF), and ILs, which regulate inflammatory responses and immune reactions (Figure 3). These factors ultimately impact the severity and prognosis of CDI by orchestrating the immune response against the infection. They recruit and activate other immune cells, facilitate bacterial clearance, and modulate inflammatory processes. However, dysregulated immune responses can contribute to CDI pathogenesis. Excessive inflammation resulting from an overactive immune response can cause tissue damage and exacerbate disease outcomes. Therefore, maintaining a balanced immune response is crucial for controlling CDI.

### Immune cells and cytokines

The innate immune system encompasses a diverse array of evolutionarily ancient hematopoietic cell types, including DCs, monocytes, macrophages, and granulocytes. Macrophages, for instance, integrate endocrine or paracrine signals with those originating from phagocytosed cells, macrovesicles, and molecules in the extracellular matrix. Furthermore, macrophages possess the ability to directly interact with surface receptors on other tissue‐resident cell populations, immune cells recruited during injury, and extracellular proteins [21].

Macrophages, along with granulocyte‐macrophage colony‐stimulating factor (GM‐CSF) and DCs, play vital roles in the immune response to CDI. They capture and process *C. difficile* antigens, migrate to draining lymph nodes, drive neutrophil recruitment in response to CDI, and activate specific T cells. Within the lymph nodes, naive T cells encounter processed antigens presented by antigen‐presenting cells, leading to their activation and differentiation into various subsets of effector T cells, including CD4^+^ T helper (Th) cells and CD8^+^ cytotoxic T cells. Once activated, these T cells migrate to the site of CDI and carry out effector functions to combat the infection. This coordinated immune response involving different cell types is essential for effectively dealing with CDI [22].

Within the intestine, ILCs play a critical role in restoring intestinal integrity postinfection. Specifically, ILC1s produce IFN‐γ and TNF, effectively curbing bacterial replication and aiding infection control [23]. ILC2s contribute to immune defense through cytokine release, including IL‐4, IL‐5, IL‐9, and IL‐33. Meanwhile, ILC3s generate cytokines such as IL‐17A, IL‐17F, IL‐22, GM‐CSF, and TNF, supporting antibacterial immunity, inflammation, or tissue restoration. The varied cytokine profiles of distinct ILC subsets collectively protect against CDI by enhancing antibacterial immunity, facilitating tissue repair, and restraining bacterial replication in the intestines [8, 24, 25, 26].

The production of IL‐17A by γδ T cells plays a crucial role in defending against CDI as it facilitates the recruitment of immune cells, enhances antimicrobial peptide production, and helps maintain gut barrier function [27]. Conversely, the absence of ILCs heightens susceptibility to CDI [8]. Mice lacking IL‐17A or γδ T cells show increased tissue inflammation and mortality from CDI. Neonatal mice with expanded RORγt^+^ γδ T cells are resistant to CDI, a resistance reversed by depleting γδ T cells or IL‐17A [28]. Additionally, IL‐33 prevents *C. difficile*‐associated mortality and epithelial disruption by promoting the activation of colonic ILC2, which in turn reduces pro‐inflammatory cytokines and increases anti‐inflammatory cytokines during CDI [29].

Natural killer (NK) cells, a distinct subset of lymphocytes originating from the bone marrow, play a pivotal role in immune defense against viral and bacterial infections, as well as cancerous proliferation. They are primarily recognized for their cytotoxic function, targeting and eliminating infected, transformed, or stressed cells [30]. Upon activation, NK cells induce cell death in target cells through various mechanisms, including the release of cytotoxic granules containing perforin and granzymes. Additionally, NK cells can produce a wide array of cytokines upon activation, including IFN‐γ, TNF‐α, and various ILs. These cytokines play a pivotal role in modulating the immune response by influencing the activity of other immune cells and shaping the overall immune environment. They can regulate inflammation, promote cell differentiation, enhance antigen presentation, and facilitate communication between different components of the immune system, thereby contributing to effective immune responses against pathogens and tumors.

IFN‐γ, for instance, stimulates the upregulation of major histocompatibility complex class II (MHC II) molecules on antigen‐presenting cells, activates macrophages, and enhances T cell response priming. Moreover, NK cells release chemokines that attract and activate crucial antigen‐presenting cells, such as DCs, to the site of tissue damage or infection. This process amplifies the immune response by recruiting additional immune cells and promoting antigen presentation, thereby strengthening immune responses against the invading pathogen [31]. Moreover, TNF‐α not only triggers the inflammatory response but also directly inhibits the proliferation of *C. difficile*, thereby mitigating infection. Conversely, IL‐10 is produced by various subtypes of immune cells, including NK cells. Notably, studies have indicated that colitis exacerbates in IL‐10‐deficient mice following CDI [32].

The cytokines produced by immune cells, including INFs, TNF, and ILs, play dual roles in CDI. Type 17 immunity, associated with cytokines like IL‐6 and IL‐23, has been implicated in severe CDI [33, 34]. IL‐17 from Th17 cells can increase mortality associated with CDI, as shown by experiments where transferring Th17 cells alone to naive mice was sufficient to increase mortality. However, in a murine model, the presence of the human commensal fungus *Candida albicans* led to a decrease in susceptibility to CDI by increasing IL‐17A expression levels [35, 36].

Understanding the complex interplay between immune cells, cytokines, and CDI is crucial for developing interventions to modulate the immune response effectively. Targeting specific immune pathways or using immunomodulatory therapies may hold promise in improving the prevention and treatment of CDI.

### Immunoregulatory factors

In the context of CDI, Toll‐like receptors (TLRs) play a pivotal role in innate immune cell recognition of specific *C. difficile* components. These receptors detect pathogen‐associated molecular patterns (PAMPs) on the bacterium's surface, initiating signaling pathways that activate nuclear factor‐kappa B (NF‐κB) and stimulate the production of pro‐inflammatory cytokines. Following activation, CD4^+^ T cells undergo differentiate into various effector subsets [37], each secreting distinct cytokines and orchestrating diverse immune responses [38]. For example, *C. difficile* flagellin activates the NF‐κB and p38 mitogen‐activated protein kinase (MAPK) pathways through TLR5, leading to IL‐8 production [39]. The interaction between CDT and TLR2 also holds significant implications for host immunity during CDI. Moreover, intact TLR2/6 signaling in mice during CDI upregulates gene pathways, including NF‐κB and MAPK [40]. Additionally, FMT modulates the TLR4 signaling pathway for CDI treatment [41]. This emphasizes the intricate interplay among microbial pathogens, host immune receptors, and therapeutic interventions in managing CDI. NF‐κB serves as a central transcription factor, governing a spectrum of innate and adaptive immune processes and acting as a key mediator in inflammatory cascades. Upon encountering CDI, NF‐κB activation ensues, prompting the expression of genes pivotal for inflammation, antimicrobial defense, and tissue regeneration. This illustrates the importance of NF‐κB in orchestrating the host response to CDI and highlights its role as a potential therapeutic target in CDI management.

Inflammasomes represent intricate multiprotein complexes that detect microbial components or cellular damage, culminating in the activation of pro‐inflammatory cytokines. This activation not only prompts the release of IL‐1β and IL‐18 but also induces a form of inflammatory cell death known as pyroptosis. The sensors of inflammasomes, including nucleotide oligomerization domain (NOD)‐like receptor family pyrin domain containing 1 (NLRP1), NLRP3, and pyrin, do not directly bind to a ligand but instead respond to specific cellular events triggered by PAMPs or danger‐associated molecular patterns. Recent research has underscored the significance of inflammasomes, particularly pyrin inflammasomes, in the host's immune response to CDI. Toxins released by *C. difficile* activate the pyrin inflammasome, initiating the processing and release of IL‐1β, thereby augmenting the inflammatory response [42, 43].

### Variability in host immune responses

The prevalence of toxigenic *C. difficile* strains varies widely, with studies suggesting that up to 71% of infants and 15% of healthy adults may asymptomatically carry these strains [44]. Research indicates that asymptomatic carriers and individuals experiencing a single episode of CDI without recurrence often exhibit stronger antitoxin immune responses compared to those with symptomatic disease [45]. Current research endeavors aim to elucidate the mechanisms underlying these differences in host immune responses, with the goal of developing more effective prevention and treatment strategies. For instance, a study demonstrated that breastfed infants with high concentrations of IgA show a protective effect against the toxin [46]. In patients with mild *C. difficile*‐associated disease, both serum levels of IgG antibody and fecal levels of IgA antibody were higher compared to those with prolonged or severe diarrhea [9]. It has been observed that the levels of antitoxin antibodies can vary significantly among both infected and uninfected individuals [47, 48], which could explain the rare occurrence of symptomatic infections despite colonization by *C. difficile*.

## POTENTIAL APPLICATION OF IMMUNETHERAPY

The defense against *C. difficile* encompasses three main lines of protection: the epithelial barrier, the rapid innate immune response, and adaptive immunity. Initially, the epithelial barrier acts as the primary defense, but it can be compromised by toxins, leading to subsequent nonspecific reactions. *C. difficile* elicits a robust pro‐inflammatory response through its virulence factors, triggering an adaptive immune reaction for effective infection management. B‐cells in GALT and lamina propria are responsible for producing IgA, which efficiently neutralizes *C. difficile* TcdA, thereby preventing its detrimental effects on host tissues. These antibodies have the capacity to directly neutralize toxins or aid in their removal from the body by enhancing phagocytosis. The humoral immune response, which involves various components such as T cells, B cells, and macrophages, integrates both innate and adaptive immune mechanisms to combat CDI. This coordinated immune response is crucial for effectively combating *C. difficile* and lowering the risk of infection [48].

### Passive immunotherapy approaches for *C. difficile* toxins

Passive immunotherapy strategies for *C. difficile* toxins entail administering specific antibodies, including monoclonal antibodies, capable of neutralizing the toxins produced by the bacterium. This approach aims to afford immediate protection and alleviate the severity of CDI by directly targeting the toxins. Consequently, it presents a promising strategy for both treating and preventing *C. difficile*‐associated diseases.

Actoxumab and bezlotoxumab (BEZ) are human‐derived monoclonal antibodies capable of individually binding to and counteracting the effects of TcdA and TcdB. BEZ, specifically, targets TcdB, thereby neutralizing its harmful effects on human cells. A study conducted between 2015 and 2019 compared the efficacy of BEZ with standard of care (SoC) therapy, typically utilizing vancomycin or fidaxomicin, to alleviate symptoms of CDI [49]. The study enrolled a total of 107 participants, with 54 in the BEZ group and 53 in the SoC group. The results showed that the incidence of recurrent CDI within 90 days was significantly lower in the BEZ group compared to the SoC group (11% vs. 43%, *p* < 0.001). Additionally, the rate of all‐cause readmission within 90 days was significantly lower for those treated with BEZ compared to SoC (40% vs. 64%, *p* = 0.011). Importantly, BEZ treatment demonstrated a significant ability to reduce the incidence of CDI without any safety concerns noted [50]. Consistent with these findings, global Phase III trials MODIFY (MOnoclonal antibodies for *C. DIFfficile* therapY) I and MODIFY II showed that BEZ effectively reduced rates of recurrent CDI compared to a placebo in individuals undergoing antibiotic treatment for CDI. It is worth noting that the administration of actoxumab, while ineffective when used alone, did not enhance the effectiveness of BEZ when administered together [49].

Traditional immunotherapy employs antibodies to counteract infection, whereas gene therapy represents an innovative paradigm, manipulating genetic material to augment immune defenses against CDI. Recently, gene therapy has been employed to achieve prolonged antibody expression. In a study, researchers engineered a novel triple mutant of adeno‐associated virus (AAV), designated as AAV6.2FF, to produce either actoxumab or BEZ in mice and hamsters. Both antibodies were detected at levels exceeding 90 μg/mL in the serum and were also observed at mucosal surfaces in both animal models. Importantly, successful expression of the antibodies was observed in all mice treated with AAV6 [51]. These findings offer a promising avenue for the development of more effective treatment approaches for CDI. By combining monoclonal antibodies such as BEZ with innovative gene therapy techniques, researchers can potentially enhance the management and prevention of CDI. This integrated approach holds the potential to improve patient outcomes and alleviate the burden of the disease by providing prolonged and targeted antibody expression, thereby offering better protection against recurrent infections.

The study conducted by Kelly et al. in 2020 provides valuable insights into the role of naturally occurring antibodies against *C. difficile* toxins, particularly TcdB, in recurrent CDI. Their research revealed that individuals with higher levels of antibodies against *C. difficile* toxin B were less likely to experience recurrent CDI [52]. This finding aligns with the results observed in the Phase 3 MODIFY trials, which evaluated the effectiveness of BEZ and actoxumab in preventing recurrent CDI. Furthermore, the observation from the MODIFY trials that administration of BEZ during CDI can prevent systemic disease and thymic atrophy without affecting gut damage is noteworthy [53]. This indicates that BEZ may exert systemic effects beyond simply neutralizing toxins within the gut. Understanding these systemic effects is essential for elucidating the full mechanism of action of BEZ and other therapeutic interventions for CDI.

The development of humanized monoclonal antibodies targeting specific toxins produced by *C. difficile* represents a promising approach in managing CDI. PA‐50 and PA‐41 antibodies, which respectively target TcdA and TcdB, have exhibited high potency in binding to specific regions of their respective toxins. In preclinical studies utilizing a hamster model of CDI, the combination therapy of PA‐50 and PA‐41 antibodies demonstrated a significantly higher long‐term survival rate compared to standard antibiotic treatment [54]. Additionally, research by Koon et al. has further elucidated the mechanism of action of these antibodies. They found that neutralizing TcdA and TcdB with human monoclonal antibodies (identified as MK3415 for TcdA and MK6072 for TcdB) effectively blocked the innate immune responses triggered by these toxins in both human colonic mucosa and human peripheral blood monocyte cells [55].

These findings underscore the therapeutic potential of targeting *C. difficile* toxins with monoclonal antibodies to mitigate the harmful effects of CDI. By neutralizing TcdA and TcdB, these antibodies may prevent the activation of inflammatory responses and tissue damage associated with CDI, ultimately improving clinical outcomes for patients.

### Passive immunotherapy approaches for sporulation

The recurrence of CDI is significantly influenced by the germination of *C. difficile* spores, which act as the primary vehicle for disease transmission. *C. difficile* can attach to the mucous layer and invade enterocytes by using proteases and flagella. This process of sporulation, triggered by nutrient scarcity, occurs during the stationary phase. Optimal conditions for spore germination in the small intestine include higher levels of cholate‐containing bile salts and lower levels of chenodeoxycholic acids, leading to the emergence of vegetative cells [56]. The interaction of *C. difficile* spores with the intestinal mucosa, critical during their penetration of the intestinal barrier, is a key factor in disease recurrence. Researchers have pinpointed specific host molecules, cellular receptors, and a spore‐surface ligand essential for the spores’ entry into intestinal epithelial cells (IECs). Notably, three collagen‐like proteins (BclA1, BclA2, and BclA3) located in the spores’ exosporium layer play significant roles [57]. BclA3, in particular, identified as an antigenic epitope, was overproduced in *Escherichia coli* and used as an immunogen in mice. The outcomes revealed that BclA3 induced specific IgG production and partially mitigated CDI symptoms following exposure to *C. difficile* spores [58]. These findings indicate BclA3's potential as an immunogen for developing a CDI vaccine.

The administration of anti‐spore IgY to C57BL/6 mice before and during CDI has demonstrated notable effects, indicating potential therapeutic benefits. First, the delay in the onset of diarrhea by 1.5 days suggests a potential slowing down of disease progression, providing a window for intervention and management. Second, a significant reduction in spore adherence to the colonic mucosa by 90% was observed, indicating a decreased colonization and invasion of *C. difficile*, which are crucial steps in the development of CDI. Furthermore, in the recurrence model, the coadministration of anti‐spore IgY with vancomycin led to a delay in recurrent diarrhea by an average of 2 days [59]. This suggests that anti‐spore IgY may have a protective effect against both initial CDI and recurrent CDI, either alone or in combination with standard antibiotic therapy. Additionally, research utilizing *Bacillus subtilis* PXN21 spores in a murine model has shown promising results. These spores activate innate immunity by upregulating the expression of TLR2, which plays a crucial role in recognizing microbial components and initiating immune responses. The activation of TLR2 leads to the release of pro‐inflammatory cytokines such as IL‐6 and TNF‐α, which can help suppress CDI symptoms and prevent both the initial occurrence and recurrence of the infection [60]. However, it is important to note that while these findings are encouraging, further research, including clinical trials in human subjects, is necessary to validate the efficacy and safety of anti‐spore IgY and *Bacillus subtilis* PXN21 spores as potential therapeutic interventions for CDI.

### Anti‐flagella

Research in both human and animal models have underscored the importance of immune responses in combating colonization and associated diseases caused by *C. difficile*. Recent attention has been directed toward understanding the role of *C. difficile* flagella in initiating immune responses. CDI development heavily relies on host colonization by *C. difficile*, with flagella playing a crucial role in various processes such as colonization, adherence, biofilm formation, and toxin production, potentially enhancing the virulence of certain strains [61]. As an intestinal pathogen, *C. difficile* utilizes flagella proteins on its surface to enhance motility, colonization, and adherence to the host intestine, thereby contributing to its pathogenicity [62]. Flagellin surface proteins, including adhesins such as FliC and flagellar cap protein FliD, along with proteases, interact with TLR5 and facilitate pathogen attachment to the mucosa [63]. This interaction triggers the activation of signaling pathways such as NF‐κB and MAPK, leading to the production of pro‐inflammatory cytokines and the initiation of an inflammatory response [64]. Research indicates that utilizing flagella‐based proteins as antigens for immunotherapy shows promise in combating CDI. Studies in mice have demonstrated that immunization with flagellin followed by CDI leads to stronger immunity toward toxins, resulting in a significant decrease in fecal presence of the pathogen [65]. Additionally, research by Pechine et al. observed that patients with active CDI exhibited lower levels of antibodies against FliC and FliD compared to a control group, suggesting a potential role for these antibodies in combating CDI [63].

### Active vaccination against CDI

Previous research highlights significant efforts in developing vaccines to combat CDI, a challenge exacerbated by antibiotic resistance and recurrent infections. Sanofi Pasteur and other research institutions are actively pursuing vaccine candidates targeting populations at risk of CDI, such as hospitalized patients or those on prolonged antibiotic regimens. Sanofi Pasteur's vaccine, intended to prevent primary CDI, is currently undergoing phase II clinical trials (NCT00772343 and NCT01230957), showing promise in reducing CDI burden in vulnerable populations [66]. Additionally, the *C. difficile* toxoid vaccine (lot number 05C02) has demonstrated safety and efficacy in phase I trials, supporting its further development for prevention purposes [67]. In Japan, a phase I trial by Inoue et al. found the *C. difficile* vaccine to be safe and well‐tolerated among healthy older Japanese individuals. Recombinant toxin‐based peptides and surface‐associated antigens remain essential vaccine candidates for future CDI prevention efforts, despite Pfizer's recent vaccine based on toxins failing in phase III clinical trials.

Furthermore, ongoing research is currently emphasizing mucosal vaccines, which could offer advantages for both adults and children, considering the critical role of mucosal immunity in fighting CDI. Recent studies indicate that developing an active, nontoxic vaccine presents a feasible strategy for preventing CDI. To this end, the CdeM protein, serving as a spore antigen, has been chosen as a carrier for mucosal immunization in vaccine development. Administration of this antigen orally has been shown to induce humoral responses against CDI in mice [68]. In the United Kingdom, Bradshaw et al. found that specific traits of lipoprotein CD0873 have potential as a vaccine against *C. difficile*. Mice given recombinant lipoprotein CD0873 showed long‐term prevention of *C. difficile* colonization in the gut due to the development of a strong secretory IgA immune response [69]. These findings have significant implications for vaccine development against *C. difficile* and may advance CDI treatment and prevention strategies.

### Gut microbiota‐elicited immune response to CDI

Gut microbiota plays a crucial role in maintaining intestinal balance and modulating immune responses. Certain beneficial bacteria in the gut can activate the immune system, stimulate the production of antimicrobial peptides, and enhance pathogen surveillance, thereby boosting overall immune function [70, 71, 72]. Interventions aimed at restoring a healthy gut microbiota, such as probiotics, FMT, or prebiotics, have shown promise in improving treatment outcomes in CDI patients, strengthening host immunity, and reducing the risk of recurrent infections.

**Figure 4:**
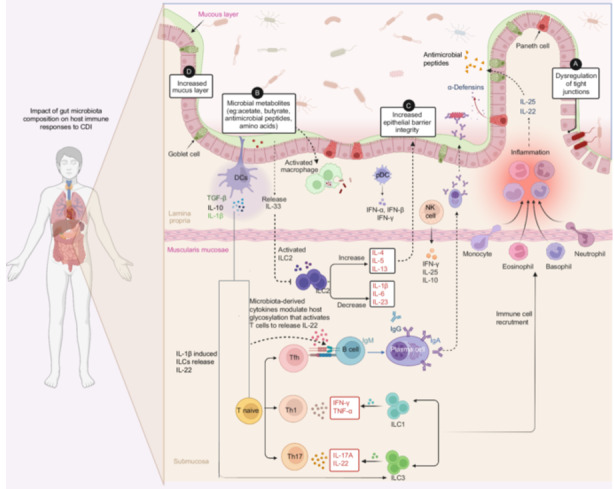
The composition of the gut microbiota profoundly influences the host immune response to CDI. In patients with CDI, compromised gut integrity facilitates the translocation of microbes across the gut barrier, leading to systemic immune dysregulation. Microbial metabolites activate immune responses by DCs and macrophages, promoting the differentiation of inflammatory T‐cell subtypes such as Th1, Th17, and Tfh cells. B cells not only produce antibodies but also coordinate the immune response against CDI. Additionally, B cells can be directly activated by the cytokine IL‐22, inducing their differentiation into plasma cells that produce protective secretory IgA (sIgA), as well as potentially pathogenic autoantibodies. The gut microbiota plays a protective role against CDI by regulating *C. difficile* growth through the production of metabolites like SCFAs, antimicrobial peptides, and amino acids. These metabolites can modulate ILCs via IL‐33. IL‐33 acts to suppress pro‐inflammatory cytokines such as IL‐1β, IL‐6, and IL‐23, while promoting anti‐inflammatory cytokines like IL‐4, IL‐5, and IL‐13. Consequently, ILC2 activation strengthens the integrity of epithelial barrier tight junctions, while ILC1s enhance local immune responses against infections through the secretion of IFN‐γ and tumor necrosis factor TNF‐α. Under the influence of IL‐1β, both ILC3s and Th17 cells can be induced to produce IL17A and IL‐22. The secretion of α‐defensins by Paneth cells and goblet cells serves as a critical source of antimicrobial peptides released into the lumen, helping to regulate the composition of luminal bacteria. Tfh, T follicular helper; SCFAs, short‐chain fatty acids; ILCs, innate lymphoid cells; NK cells, natural killer cells; pDC, professional dendritic cell.

The intestinal innate immune system coordinates a diverse array of defensive mechanisms against CDI. This intricate defense involves a sophisticated interplay among multiple components aimed at preserving intestinal integrity and homeostasis (Figure 4). Key constituents include IECs, macrophages, monocytes, mast cells, ILCs, and DCs [73]. IECs line the intestinal mucosa, forming a physical barrier against pathogens and toxins. They encompass absorptive enterocytes responsible for nutrient absorption and barrier function, goblet cells producing protective mucus, Paneth cells secreting antimicrobial peptides, and enterochromaffin cells producing serotonin and influencing gut motility and immune responses. The immune cells involved in intestinal innate immunity include macrophages, which are phagocytic cells engulfing pathogens and cellular debris, monocytes circulating and capable of differentiation into macrophages or DCs, mast cells releasing histamine and other mediators in response to pathogens or allergens, and ILCs, such as ILC1s, ILC2s, and ILC3s, contributing to immune regulation, tissue repair, and defense against infections. Additionally, DCs function as antigen‐presenting cells regulating adaptive immune responses.

ILCs, notably, play a pivotal role in restoring intestinal integrity postinfection [24]. Stimulation of ILCs by microbiota prompts cytokine release, recruiting the host immune system to combat infection. Microbiota‐derived acetate has been demonstrated to enhance innate responses to *C. difficile* by synergistically modulating neutrophils and ILC3s. This modulation facilitates inflammasome activation in neutrophils and increases IL‐1 receptor expression in ILC3s, ultimately boosting IL‐22 secretion in response to IL‐1β to counteract CDI [74].

Microbiota‐derived cytokines play a pivotal role in safeguarding the mucosal barrier to against CDI. IL‐17 and IL‐22, secreted by γδ T cells in the small intestine lamina propria, are stimulated by the gut microbiota [36]. Activation of mucosal IL‐22 signaling, facilitated by gut microbiota colonization, modulates host N‐linked glycans' glycosylation, promoting the growth of bacteria that consume succinate, such as *Phascolarctobacterium spp*. This metabolic alteration prevents the proliferation of *C. difficile* in the gut [75, 76]. FMT has proven to be a successful therapeutic strategy for recurrent and severe CDI. FMT has been found to restore the gut microbiota composition, leading to increased levels of cytokines and antibodies. Successful FMT therapy correlates with heightened proportions of TcdB‐specific Th17 cells, crucial for combating *C. difficile* toxins. Moreover, increased levels of IgG and IgA antibodies targeting TcdA and TcdB are observed post‐FMT, pivotal for neutralizing *C. difficile* toxins [77]. Studies indicate elevated levels of IL‐25, a microbiota‐derived cytokine, in colonic tissue post‐FMT. IL‐25 mitigates CDI‐associated mortality and tissue damage by recruiting eosinophils and selectively reducing harmful IL‐23, thereby modulating the inflammatory response [78]. Treatment with IL‐33 dampens the inflammatory response linked to CDI by reducing pro‐inflammatory cytokines such as IL‐1β, IL‐6, and IL‐23, while boosting anti‐inflammatory cytokine production like IL‐4, IL‐5, and IL‐13. IL‐33's protection against CDI hinges on the presence of ILC2s, which produce IL‐13 [29]. IL‐13 is vital for recruiting or transitioning these cells into macrophages. Neutralizing the decoy receptor IL‐13Rα2 confers protection from CDI, underscoring the significance of IL‐13 signaling in defending against the infection [79].

The human gut microbiota plays a pivotal role in synthesizing essential metabolites that profoundly impact human health. Short‐chain fatty acids (SCFAs), including acetate, propionate, and butyrate, are among the most well‐studied metabolites known for their roles in immune homeostasis and host health. SCFAs are produced through the fermentation of dietary fibers and complex carbohydrates by gut bacteria, and they offer multiple beneficial effects. These include stimulating intestinal IgA responses and facilitating intercommunication between the host and microbiome [80]. Additionally, they regulate colonic pH to maintain immune homeostasis and modulate proliferation, differentiation, and gene expression in mammalian colonic epithelial cells [81, 82, 83]. Acetate, one of the SCFAs, enters the systemic circulation and exerts immunomodulatory effects on different immune cells such as monocytes, T cells, and neutrophils. It influences the balance between antioxidants and oxidants and affects cytokine production in these immune cells [84]. Butyrate, on the other hand, demonstrates anti‐inflammatory properties by inhibiting pro‐inflammatory cytokines and reducing leukocyte recruitment to inflammatory sites. Moreover, it enhances the population of regulatory T cells and promotes the development of tolerogenic CD103^+^ DCs, thus alleviating intestinal inflammation and enhancing intestinal barrier function, as evidenced in CDI mice [85].

Beyond SCFAs, the gut microbiota contributes to synthesizing amino acids, vitamins, and secondary bile acids, all of which play crucial roles in host physiology and immune function. For instance, certain gut bacteria produce vitamins such as vitamin K and B vitamins, essential for various metabolic processes and immune function. Secondary bile acids, resulting from the biotransformation of primary bile acids by gut bacteria, are implicated in regulating host metabolism and immune responses.

## CONCLUSION

The host immune response plays a pivotal role in combating CDI, a pathogenic bacterium known for causing gastrointestinal infections. Through a myriad of mechanisms such as producing antimicrobial peptides, engulfing and destroying pathogens, and regulating inflammatory responses, the immune system identifies, eliminates, and prevents infections triggered by this bacterium. Additionally, the immune system fosters the proliferation of beneficial gut bacteria to uphold intestinal homeostasis and mitigate the likelihood of CDI occurring or recurring.

Hence, fortifying the host immune response stands as a cornerstone in both preventing and treating CDI. By comprehending and fostering the interaction between the immune system and gut microbiota, we can craft more effective strategies for prevention and control.

## AUTHOR CONTRIBUTIONS

Lamei Wang, Xinhua Chen and Yangchun Cao conceived the idea and edited the manuscript. Lamei Wang, Javier A. Villafuerte Gálvez and Christina Lee collected the data and wrote the manuscript. Lamei Wang and Yangchun Cao drew the figures. Javier A. Villafuerte Gálvez and Ciaran P. Kelly validated and supervised the manuscript. All authors have read the final manuscript and approved it for publication.

## CONFLICT OF INTEREST STATEMENT

The authors have declared no competing interests.

## ETHICS STATEMENT

No new animal or human experiments were involved in this study. All data were obtained from publicly available research and comply with ethical standards.

## Data Availability

Data sharing not applicable to this article as no datasets were generated or analyzed during the current study. No new data and scripts were generated in this review. Supplementary information (graphical abstract, slides, videos, Chinese translated version, and update materials) are available online DOI or http://www.imeta.science/.
